# Clinical and epidemiological profile of infants with severe bronchiolitis before and after the COVID-19 pandemic

**DOI:** 10.1016/j.jped.2026.101595

**Published:** 2026-09-15

**Authors:** Gustavo F. Wandalsen, Bárbara C.F. Ramos, Dirceu Solé, Douglas A.S.M. Miotto, Ivan Rondelli, Bruna C.M. Almeida, Flávia C. Pontes, Mayumi F. Wada, Juliana Franceschini, Angela Honda, Daniella G.B.P. da Silva, Regina G. Cesar

**Affiliations:** a Instituto Pensi, São Paulo, SP, Brazil; b Universidade Federal de São Paulo (UNIFESP), São Paulo, SP, Brazil; c Sabará Hospital Infantil, São Paulo, SP, Brazil; d Fundação ProAR, Salvador, BA, Brazil; e Faculdade de Ciências Médicas da Santa Casa de São Paulo, São Paulo, SP, Brazil

**Keywords:** Bronchiolitis, Respiratory syncytial viruses, COVID-19, Infant

## Abstract

**Objective:**

To describe clinical and epidemiological characteristics of infants hospitalized with severe Acute Viral Bronchiolitis (AVB) before and after the COVID-19 pandemic and to identify factors associated with intensive care unit (ICU) admission.

**Methods:**

This retrospective study analyzed medical records of infants aged 1–24 months hospitalized for AVB at a private pediatric hospital in São Paulo, Brazil, in 2018–2019 and 2022–2023. Demographic and clinical variables were evaluated. Logistic regression was used to identify factors associated with ICU admission.

**Results:**

A total of 2363 infants were included; 55.5% were male, and 56.7% were ≤6 months old. ICU admission was required in 51.8% of cases, and 45.3% required intensive respiratory support. RSV was identified in 44% of cases and was associated with greater severity, including higher ICU admission rates, increased need for intensive respiratory support, longer hospital stays, and more complications. Independent risk factors for ICU admission included RSV infection (OR 2.18; 95%CI: 1.8–2.6), age ≤ 6 months (OR 2.92; 95%CI: 2.4–3.5), oxygen saturation ≤ 93% at admission (OR 2.48; 95%CI: 2.0–3.0), and relevant personal history (OR 1.30; 95%CI: 1.1–1.6). Post-pandemic, infants were younger, had higher ICU admission rates, lower RSV detection, fewer complications, and increased corticosteroid use. Seasonal patterns were also altered, with an additional incidence peak observed in spring.

**Conclusions:**

RSV infection, younger age, and hypoxemia at admission were independently associated with ICU admission in infants hospitalized with severe AVB. Changes observed in the post-pandemic period included shifts in seasonality, patient profile, and management patterns.

## Introduction

Acute viral bronchiolitis (AVB) is the most common lower respiratory tract infection and one of the leading causes of hospitalization and death in children under one year of age. The clinical presentation is highly variable, ranging from nonspecific signs and symptoms to mild to severe respiratory distress, and severe AVB is commonly defined as those requiring hospitalization [1,2]. Generally, the criteria for hospital admission are respiratory distress, low saturation at admission, dehydration, and/or apnea. Respiratory syncytial virus (RSV) is the primary cause of 60% to 80% of cases [3].

Analysis of data from national health systems has provided further insight into the impact of AVB on patients’ lives. According to a Canadian study, hospitalizations due to AVB accounted for 13% of all-cause hospitalizations in children in that country [3]. Studies in Brazil using data from the Unified Health System (SUS; 2000–2019) showed that 57% of hospitalizations due to AVB occur in children under one year of age, with boys being the most affected, as well as residents of the southeastern area [4,5].

When assessing the impact of specific RSV infections, the data are even more dramatic. A study of Italian children hospitalized for RSV infection (2011–2020) showed that 75% of them were under one year of age; more specifically, 49.6% were under two months old, and 25% had severe forms of the disease. RSV was responsible for 48.9% of cases of acute respiratory failure in children under one year of age [6].

A systematic review assessed the impact of RSV infections in children and adults across several Latin American countries (between 2012 and 2023), with 25% of the studies conducted in Brazil. Of the total, 73.7% of hospitalizations were in children, and 58% were in children under one year of age. Although the rate of admission to the Intensive Care Unit (ICU) was high (42%), the case-fatality rate for acute RSV infection was low, at 0.6% in patients under two years of age and 3% in those up to five years of age [7].

Age under two months of age, longer hospital stay, history of prematurity, presence of congenital heart disease, and exposure to tobacco smoke and air pollution have been identified as risk factors for increased severity of AVB [6,8].

In 2020, with the emergence of severe acute respiratory syndrome caused by Coronavirus 2 (SARS-CoV-2) and the implementation of several measures to contain the virus, there was a clear impact on the circulation of other respiratory viruses, including RSV. Changes in the seasonality of respiratory viruses and in the clinical profile of affected infants have been reported, but only a few from Brazil.

In this study, the authors evaluated a large sample of infants with severe AVB at a private hospital in São Paulo, Brazil, aiming to describe their characteristics and identify possible risk factors for poor outcomes and the need for ICU admission. Data were collected over four years, two before the COVID-19 pandemic and two during and after the pandemic.

## Methods

This is a retrospective and observational study based on data collected from electronic medical records, available in the MV SOUL system, of patients admitted to Sabará Children’s Hospital, with a diagnosis of AVB. Severe AVB was considered an admission to the hospital regardless of the unit. Based on hospital protocols, patients were hospitalized if they presented with low oxygen saturation and respiratory distress that did not improve after initial management or had any difficulty in feeding. Patients aged 1 to 24 months, regardless of sex, admitted for AVB during the years 2018, 2019, 2022, and 2023 with a diagnostic code at admission or discharge that included at least one of the following ICD-10 codes: J21, J21.0, or J21.8, were included. Patients with chronic lung disease, those with a prior diagnosis of wheezing, bronchiolitis, wheezing infants, or those using inhaled corticosteroids, or those who had already undergone any prophylactic treatment and/or follow-up for wheezing were excluded. Patients transferred to another hospital before discharge were also excluded.

Patients were evaluated according to the following variables: age, sex, relevant personal history (prematurity, twinning, and heart disease), vital signs on admission, need for admission to the ICU, need for oxygen therapy, and type of respiratory support used [simple nasal oxygen catheter, Venturi mask, high-flow nasal cannula (HFNC), noninvasive ventilation (NIV), and mechanical ventilation (MV)—the latter three defined as “intensive respiratory support”], use of antibiotics and systemic corticosteroids during hospitalization, presence of complications or other associated infections, and length of stay. Patients were evaluated for RSV infection using a PCR viral panel and/or rapid antigen test.

For case classification, any patient testing positive for RSV — whether through viral panel or rapid antigen test — was considered to have an RSV-associated infection. Abnormal peripheral oxygen saturation (SaO₂) was defined as ≤ 93%. A complication during hospitalization was defined as the presence of pneumonia, atelectasis, pneumothorax, or acute otitis media documented in the medical record. Regarding oxygen support, the most intensive type of support used was recorded.

Continuous variables were presented as mean and standard deviation, and categorical variables as percentages. Univariate comparative analysis was performed using the chi-square test to assess associations between categorical data and Student’s *t*-test for continuous variables. Variables with *p* < 0.20 in univariate analysis and/or considered clinically relevant were included in the multivariate logistic regression model to identify independent predictors of ICU admission. A *p*-value < 0.05 was considered statistically significant. The results were presented as odds ratios (OR) with 95% confidence intervals (95%CI).

The study was approved by the Research Ethics Committee (# 6.949.579, #6.195.199), and the requirement for an Informed Consent Form was waived.

## Results

During the study period, 384,304 patients were evaluated in the emergency department. Of this total, 3248 met the inclusion criteria, and 885 were excluded mainly due to a previous episode of wheezing and/or prior respiratory disease. So, 2363 cases were included in the study, distributed as follows: 2018: 598 included among 102,133 visits (0.59%); 2019: 606 included among 100,570 visits (0.60%); 2022: 524 included among 94,091 visits (0.56%); and 2023: 635 included among 87,510 visits (0.73%). Most of these patients were male (55.5%), and 57% were aged six months or younger. ICU admission was required in 52% of cases, and intensive respiratory support was used in 45%. RSV etiology was tested in 2333 infants and was identified in 44% of infants. Among all the patients included, none died during hospitalization. Clinical and demographic characteristics of infants with severe AVB according to etiology (RSV or non-RSV) are shown in Table 1.

**Table 1:** Clinical and demographic characteristics of infants with severe acute viral bronchiolitis (N: 2363) stratified by viral etiology (RSV or non-RSV).

Characteristic	Acute viral bronchiolitis			*p*
	Total - *N* (%)	RSV#		
		Yes 1032 (%)	No 1301 (%)	
**Age ≤ 6 months**	1340 (56.7)	438 (42.4)	594 (45.7)	0.56
**Male**	1313 (55.5)	561 (54.3)	757 (58.2)	0.04
**Dyspnea**	1971 (83.4)	885 (85.8)	1061 (81.6)	0.006
**Wheezing**	1644 (69.5)	753 (73.0)	866 (66.6)	0.001
**Tachypnea**	1458 (61.7)	663 (64.2)	771 (59.3)	0.01
**SaO_₂_≤ 93% on admission**	1057 (44.7)	513 (50.9)	513 (39.4)	<0.001
Relevant personal history*	634 (26.8)	251 (24.3)	376 (28.9)	0.02
**ICU**	1224 (51.8)	640 (62.0)	574 (44.1)	<0.001
**Intensive respiratory support**⁎⁎	1071 (45.3)	575 (55.7)	488 (37.5)	<0.001
**Antibiotic use**	1053 (44.5)	485 (47.0)	557 (42.8)	0.04
**Any complications**##	846 (35.8)	393 (38.1)	440 (33.8)	0.03
**Pneumonia**	358 (15.1)	193 (18.7)	161 (12.4)	<0.001
**Sepsis**	78 (3.3)	47 (4.6)	30 (2.3)	0.003
**Atelectasis**	161 (6.8)	92 (8.9)	67 (5.1)	<0.001
**Length of hospital stay**	6.2 ± 4.7	7.0 ± 5.0	5.7 ± 4.4	<0.001
**Days in the ICU**	3.7 ± 4.7	4.8 ± 4.9	2.8 ± 4.4	<0.001

O_2_, oxygen.

# Rapid test and/or PCR, N:2333 (RSV test was not available in 30 cases).

⁎ Prematurity, twinning, heart disease, or severe illness.

⁎⁎ High-flow catheter, noninvasive ventilation, or mechanical ventilation.

## Pneumonia, sepsis, pneumothorax, atelectasis.

Identification of RSV infection was associated with greater initial severity of the AVB at admission, a higher frequency of wheezing, tachypnea, and abnormal SaO₂ (Table 1). Infants with RSV-related AVB also required ICU admission and intensive respiratory support more frequently, had higher antibiotic use, higher frequency of complications, and longer hospital and ICU stays (Table 1).

**Figure 1:**
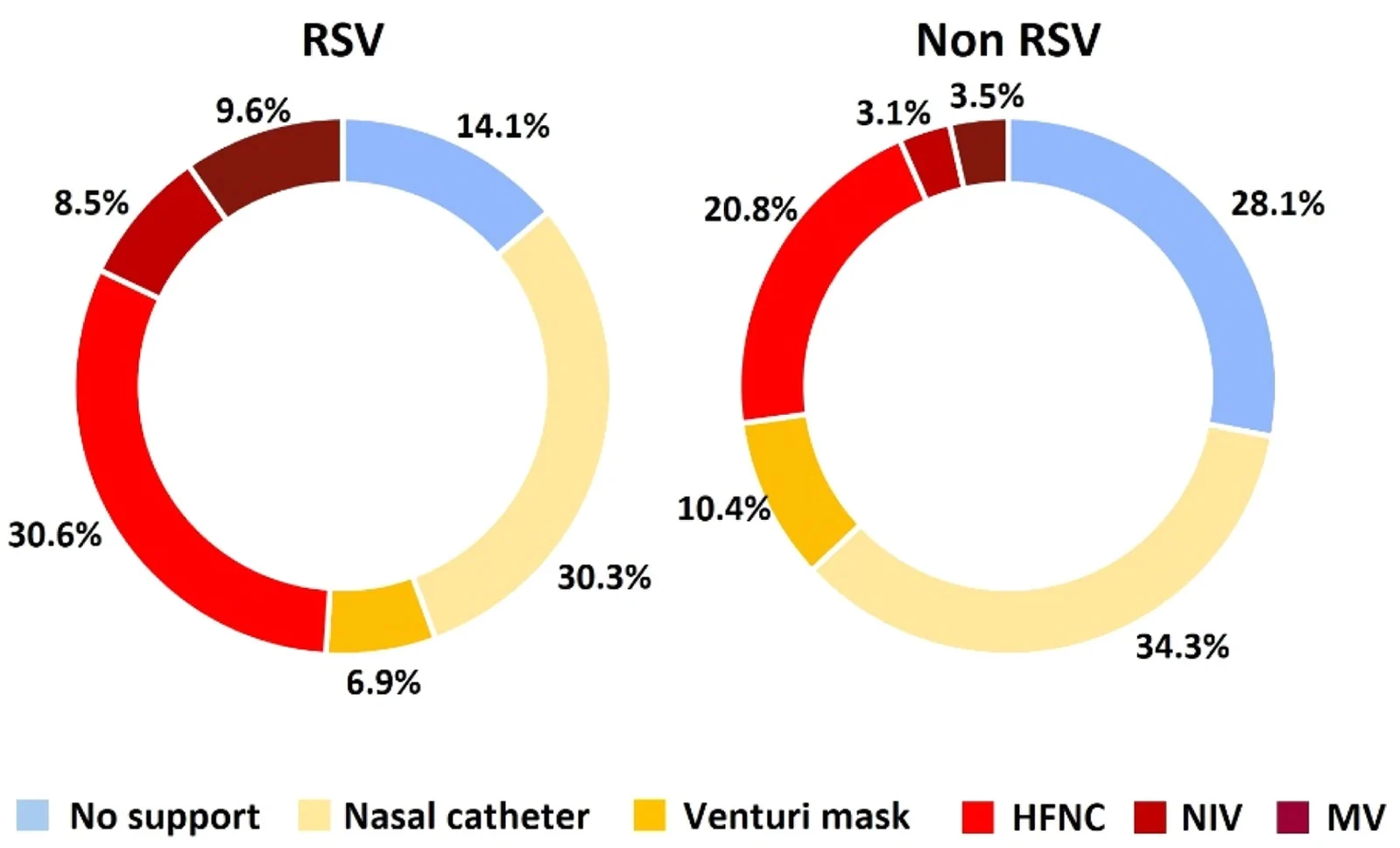
Respiratory support used in infants with severe bronchiolitis classified by viral etiology (RSV or Non-RSV).

HFNC: high-flow nasal cannula; NIV: noninvasive ventilation: MV: mechanical ventilation.

Of the total patients, 1834 (77.6%) required oxygen support, with the predominant use of simple oxygen catheters (32.7%) and HFNC (25.1%), followed by Venturi masks (8.7%), MV (6.1%), and NIV (5.4%). More intensive forms of respiratory support were significantly more frequently used among infants with RSV-AVB, as shown in Figure 1.

ICU admission was required in 1224 cases (51.8% of the total). RSV-AVB, age under six months, abnormal SaO₂ at admission, relevant personal history and study period (pre- or post-pandemic) were identified as factors significantly associated with ICU admission (Table 2).

**Table 2:** Factors associated with ICU admission of infants with severe acute bronchiolitis identified by binary logistic regression.

	Odds ratio	95% Confidence interval	*p*
**RSV**#	2.18	1.8 – 2.6	<0.001
**Age ≤ 6 months**	2.92	2.4 – 3.5	<0.001
**SaO_₂_≤ 93% on admission**	2.48	2.0 – 3.0	<0.001
**Relevant personal history**##	1.30	1.1 – 1.6	0.009
**Study period**###	2.45	2.0 – 2.9	<0.001

Factors included in the analysis: RSV, age ≤ 6 months, SaO_2_ ≤ 93%, relevant personal history, study period and sex.

# Rapid test and/or PCR.

## Prematurity, twinning, and heart disease.

### 2022–2023 *vs* 2018–2019.

A comparison between the groups evaluated before (2018 and 2019) and after the COVID-19 pandemic (2022 and 2023) revealed a decrease in the proportion of male infants, an increase in infants aged six months or younger, and a decrease in the proportion of RSV identification via viral panel and/or rapid antigen test. Despite a higher frequency of ICU admission in the post-pandemic years, there were no differences in length of hospital stay, and a reduction in the frequency of complications was observed. Regarding treatment, the use of systemic corticosteroids was twice as high in the post-pandemic years; no differences were observed in antibiotic use (Table 3).

**Table 3:** Clinical and demographic data of infants with severe bronchiolitis before (2018–2019) and after the onset of the COVID-19 pandemic (2022–2023).

Feature	2018–2019 *N* = 1204 (%)	2022–2023 *N* = 1159 (%)	*p*
**Male**	708 (58.8)	625 (53.9)	0.02
**Age ≤ 6 months**	623 (51.7)	717 (61.9)	<0.001
**Wheezing**	898 (74.6)	747 (64.5)	<0.001
**SaO_₂_ ≤ 93% on admission**	658 (54.7)	399 (34.4)	<0.001
**Relevant personal history**	330 (27.4)	304 (26.2)	0.21
**RSV**#	562 (46.7)	470 (40.6)	<0.001
**ICU**	522 (43.4)	702 (60.6)	<0.001
Intensive respiratory support*	533 (44.3)	538 (46.4)	0.32
**Use of corticosteroids**	135 (11.2)	256 (22.1)	<0.001
**Antibiotic use**	554 (46.0)	499 (43.1)	0.16
**Any complications**##	477 (39.6)	369 (31.8)	<0.001
**Length of hospital stay**	6.1 ± 4.6	6.3 ± 4.8	0.32

# Rapid test and/or PCR.

⁎ High-flow catheter or noninvasive ventilation or mechanical ventilation.

## Pneumonia, sepsis, pneumothorax, atelectasis.

**Fig. 2:**
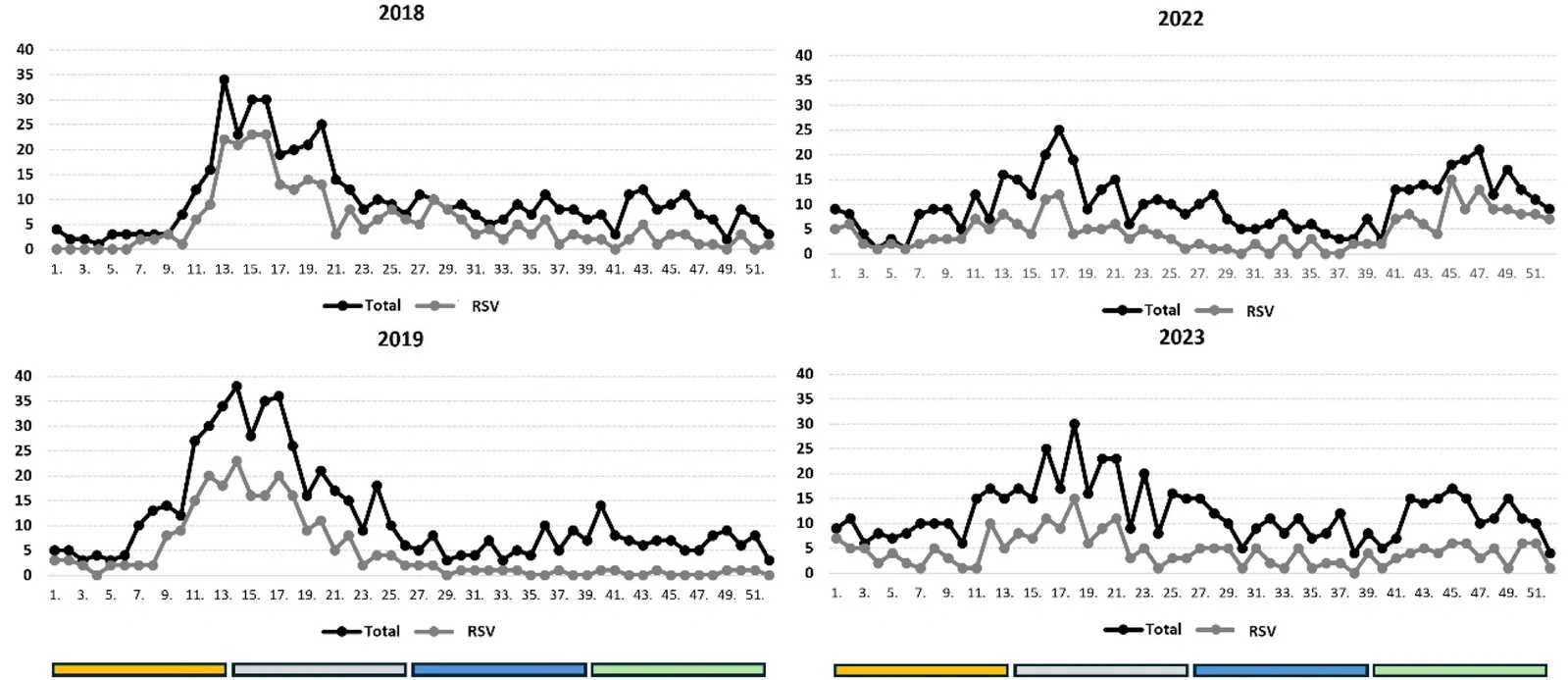
Weekly incidence of severe bronchiolitis cases (total cases and RSV-related) in the years 2018, 2019, 2022, and 2023.

The distribution of cases by epidemiological week over the four years of the study is shown in Figure 2. The most typical seasonal pattern, characterized by peaks in case incidence between weeks 13 and 19 (April to May), is observed in 2018 and 2019 but is clearly altered in 2022 and 2023. In these years, a less prominent peak is observed in the fall and a second peak in incidence between weeks 42 and 48 (October and November). In cases of RSV-AVB, the incidence curve tends to follow the total cases curve, except in 2019, when cases occurred predominantly in the first semester of the year, during the typical seasonal period (Figure 2).

Summer: yellow; Fall: gray; Winter: blue; Spring: green.

## Discussion

In the present study, the authors present data from a large cohort of infants with severe AVB hospitalized at a private pediatric hospital in São Paulo, Brazil. Classic characteristics of severe bronchiolitis associated with hospitalization were observed: male predominance, younger infants, and presence of comorbidities (Table 1).

The hospitalization rate among patients ranged from 0.56% in 2018 to 0.73% in 2023, reflecting a modest increase, consistent with observations reported by other studies in patients under two years of age [3,9].

RSV is widely recognized as the leading cause of severe acute respiratory infections in infants, especially during the first year of life. During the acute phase, infants with RSV-associated acute respiratory infections have been reported to exhibit more severe symptoms [2,3,10], a finding observed in the present study. In addition to a higher rate of ICU admission (62% vs. 44%), several other markers of severity were found, such as longer hospital stays, higher rates of complications and antibiotic use (Table 1), as well as the use of more intensive respiratory support therapies (Figure 1). In the multivariate analysis, the presence of RSV-AVB was associated with a twofold increased risk of ICU admission (Table 2).

The relevance of RSV as a causative agent of respiratory infections in infants extends beyond severe cases of bronchiolitis. Following hospital discharge, prospective studies indicate a greater need for healthcare services and higher costs to the healthcare system among children who had RSV-AVB [[11], [12], [13]]. RSV-AVB is also recognized as a risk factor for recurrent wheezing in preschool-aged children and for asthma in school-aged children [14].

In a context where little progress has been made in preventing severe bronchiolitis in recent decades, the development of an RSV vaccine for pregnant women and a new monoclonal antibody can be considered a game-changer. Findings from various parts of the world indicate a drastic change in the landscape of severe bronchiolitis following the implementation of these immunization strategies [15,16], a situation not yet captured in the present study, which was conducted before the development of these immunizers.

Hypoxemia is a common finding in severe bronchiolitis, resulting from airway obstruction and atelectasis. High-quality evidence identifies hydration and oxygenation as key elements in the management of AVB [10]. Among intensive respiratory support strategies, the use of HFNC was employed in 25% of infants in this study, a rate substantially higher than that observed in those undergoing NIV and MV. The hospital’s respiratory support strategy provides for a stepwise use of the oxygen modalities, with HFNC (1–2 L/kg/min) mainly reserved for cases in which hypoxemia cannot be adequately controlled with a simple oxygen catheter and/or a Venturi mask.

High-quality evidence regarding the role of HFNC in AVB is hampered, in part, by the diversity of HFNC application protocols (14). Several studies do not demonstrate the superiority of HFNC over low-flow oxygen modalities [17,18]. Despite this, guidelines and reviews frequently recommend HFNC in cases of failure of low-flow modalities [10,19,20]. Another point of discussion concerns the HFNC’s ability to prevent the progression of severe AVB cases to more intensive modes of respiratory support. In the present study, 865 infants received oxygen via HFNC, and of these, 272 (31.4%) required progression to NIV and/or MV.

In Brazil, the seasonal pattern of RSV begins in the fall (late March), peaks in April and May, and declines by early winter (July), with minor variations possible across the country’s geographic regions [21]. In our cohort, it was observed that in the post-pandemic years (2022/23), there was a lower peak of bronchiolitis cases during the usual seasonal period (fall), with the emergence of a second wave in October and November (spring).

Several countries reported a delay in RSV seasonality during the pandemic period, including Brazil [22], Argentina, and the Dominican Republic [23], as well as Canada, Japan, and Korea [24]. In Italy, for example, following a disruption in the usual seasonal pattern, the subsequent seasonal wave occurred earlier than expected [25,26]; meanwhile, in the United States, RSV positivity remained constant throughout the pandemic [27].

In addition to shifts in seasonality, changes were also observed in the clinical characteristics of patients admitted with bronchiolitis in the pre- and post-pandemic periods. In 2022/23, hospitalized infants were younger, were more frequently admitted to the ICU, and received more systemic corticosteroids. Furthermore, the frequency of RSV identification was lower. A study conducted in Italy also showed younger patients hospitalized for bronchiolitis after the pandemic, but unlike our cohort, RSV accounted for about 80% of hospitalized cases [21]. Discrepancies from the present findings were also reported in another Italian study, which observed a higher median age and a greater proportion of mild bronchiolitis cases in the post-pandemic period [25].

The higher ICU admission rate in the post-pandemic years was identified as an independent factor in logistic regression. This observation may not directly indicate greater disease severity but rather be associated with changes in medical practices and in patient characteristics, such as the younger age of the infants. The reduction in complication rates and the stability of antibiotic prescription rates and length of hospital stay in the years 2022–23 support this hypothesis. It's important to point out that the bronchiolitis criteria for ICU admission haven't changed during the years of the study and included: age ≤ 2 months, respiratory failure (Wood-Downes ≥ 4), need for respiratory support with HFNC, NIV, or MV, and clinical instability (PEWS ≥ 4).

Systemic corticosteroids are not routinely recommended for the treatment of severe bronchiolitis [19]. The authors can speculate on a few reasons for the noticeable increase in the prescription rate of systemic corticosteroids observed in the years after the pandemic. During the pandemic, systemic corticosteroids were used in cases of severe acute respiratory syndrome caused by SARS-CoV-2. Additionally, some recent studies have suggested that using systemic corticosteroids in cases of rhinovirus bronchiolitis might delay the future onset of wheezing episodes [28].

The present study has some limitations. Clinical and follow-up data were retrospectively extracted from medical records, with the possibility of unrecorded information. Not all patients underwent viral panel testing by PCR; etiologic information regarding RSV was the most reliable. The sample was obtained from a single private hospital that does not serve patients from the public health system (SUS) and may not be representative of São Paulo’s general population.

In conclusion, consistent with findings in the literature, RSV was identified as a factor associated with greater severity in hospitalizations due to acute bronchiolitis, as well as younger age and the presence of signs of hypoxemia at hospital admission. Oxygen support was required in the vast majority of cases, predominantly using less invasive modalities, such as HFNC. Compared to pre-pandemic years, changes were observed in the seasonal distribution of cases, a reduction in the age of infants, and changes in treatment patterns, with a higher rate of ICU hospitalization and increased use of systemic corticosteroids.

## Funding sources

None.

## Conflicts of interest

The authors declare no conflicts of interest.

## Data Availability

The data that support the findings of this study are available from the corresponding author.
